# The *v ^24^* Allele is a Missense Mutation Within the Predicted Tryptophan 2,3-dioxygenase Protein Domain of *vermilion*

**DOI:** 10.17912/micropub.biology.001760

**Published:** 2025-08-18

**Authors:** Lorielle M. Raab, Steve Kucera, Brian Oliver, Leif Benner

**Affiliations:** 1 Department of Biology, University of Tampa, Tampa, Florida, United States; 2 Section of Developmental Genomics, Laboratory of Biochemistry and Genetics, National Institutes of Health, Bethesda, Maryland, United States; 3 Unit on Chromosome Dynamics, Division of Developmental Biology, Eunice Kennedy Shriver National Institute of Child Health and Human Development, National Institutes of Health, Bethesda, Maryland, United States

## Abstract

Many loss-of-function mutations in the *Drosophila* *vermilion* ( *v* ) gene have been described. However, the causal mutation in the common *v ^24^* allele is unknown. We sequenced different *v* alleles ( *v ^24^* , *v ^+^* , and *v ^1^* ) to identify candidate *v ^24^* mutations. We identified a single T>A missense mutation shared among the three *v ^24^* chromosomes, resulting in a Phe>Ile amino acid change within the predicted tryptophan 2,3-dioxygenase protein domain. This same T>A missense mutation has been independently shown to result in a *v ^–^* phenotype by Nivard et al., 1993 and is therefore strong corroborating evidence that this mutation is causal for the *v ^–^* phenotype in the *v ^24^* allele.

**Figure 1. Candidate vermilion SNPs identified from DNA-sequencing.**
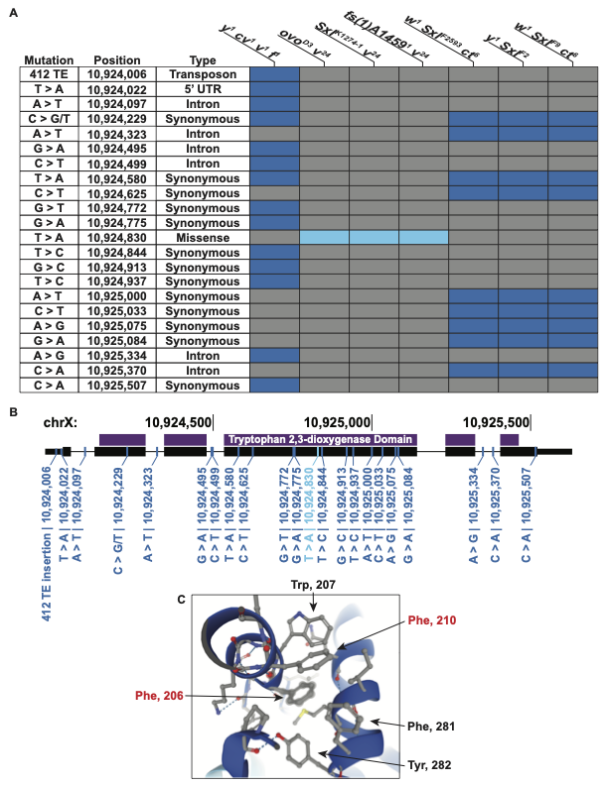
A) List of all 5’ UTR, intronic, synonymous, and missense mutations we identified from our SNP analysis. Gray boxes indicate the SNP is not present and dark blue boxes indicate the presence of a SNP along a non- *v ^24^* chromosome. Light blue boxes indicate *v ^24^* specific SNPs. B) Location of SNPs from Figure 1A along the *v* gene body. Small black boxes indicate untranslated regions, large block boxes indicate open reading frames, and purple boxes indicate the predicted tryptophan 2,3-dioxygenase protein domain. C) Predicted Alphafold structure of the region containing the putative *v ^24^* mutation. Aromatic amino acids within this region and their position along the V open reading frame are indicated. Red lettering denotes mutable amino acids within this region resulting in *v ^–^* phenotypes.

## Description

Chromosomes containing recessive mutations at the X-linked *vermilion* ( *v* ) locus are a commonly used research tool in *Drosophila* genetics. Flies mutant for *v* fail to synthesize brown pigments in the eye, resulting in a lighter red eye color (Baglioni, 1960; Baillie & Chovnick, 1971; Tartof, 1969). Phenotypic markers like *v* alleles are essential tools for *Drosophila* transgenesis because they allow for quick and accurate identification of transformants. For example, in ΦC-31 mediated transgenesis (Bischof et al., 2007), *v ^+^* duplications (Fridell & Searles, 1991) are cloned alongside transgenes of interest in a single plasmid and injected into *v ^–^* embryos (Ni et al., 2008). Screening for rescue of the v ^+^ eye phenotype can then be used to recover individuals with successful integration of the plasmid DNA. Currently, there are over 28,000 individually maintained stocks that contain *v* mutations (Öztürk-Çolak et al., 2024). The number of *v* stocks is largely due to the Transgenic RNAi Project and CRISPR collections that have been generated for the *Drosophila* research community (Zirin et al., 2020).

In addition to the original *v ^1^* allele (Morgan, 1916), other *v* alleles have also been generated and used extensively in *Drosophila* research. For example, a chromosome containing a *v ^24^* allele has been used in two independent X-linked female sterile screens (Gans et al., 1975; Komitopoulou et al., 1983). *v ^24^* was originally isolated as an EMS induced mutation and presumably, based on the authors description in two of their related works, is a null allele (Gans et al., 1975; Komitopoulou et al., 1983). However, a third work by the same research group described *v ^24^* as ‘thermosensitive’, without further details on the nature of the temperature sensitivity, possibly indicating that *v ^24^* is a hypomorphic allele (Busson et al., 1983). Regardless, EMS mutagenesis generally produces GC to AT transition mutations (Snow et al., 1984), however, other types of mutations such as transversion mutations (AT to TA and TA to GC) can also be recovered at a lower frequency (Arrizabalaga & Lehmann, 1999). Although the *v ^24^* allele exists in 57 currently maintained *Drosophila* stocks to date (Öztürk-Çolak et al., 2024), the nature of the *v ^24^* mutation has never been described. Through our work on the genetics of female fertility in *Drosophila* , we have whole genome sequencing data for a number of different X chromosome genetic backgrounds, including those bearing *v ^1^* or *v ^24^* alleles, along with those bearing wild type *v* alleles. Using our DNA-sequencing datasets, we wanted to identify a candidate causal mutation for the *v ^24^* allele.

In order to identify candidate mutations for the *v ^24^* allele, we identified all single nucleotide polymorphisms (SNPs) in the *v* gene for a single *v ^1^* chromosome ( *y ^1^ cv ^1^ v ^1^ f ^1^* ), three independent *v ^24^* chromosomes ( *fs(1)A1459 ^1^ v ^24^* , *Sxl ^K1274-1^ v ^24^* , *ovo ^D3^ v ^24^* ), and three *v ^+^* chromosomes ( *w ^1^ Sxl ^F2593^ ct ^6^* , *y ^1^ Sxl ^F2^* , *w ^1^ Sxl ^F9^ ct ^6^* ). Our SNP analyses cumulatively identified 21 different SNPs and 1 transposable element insertion for all 7 sequenced *v* alleles ( Figure 1A, B). We confirmed the unique *412* transposable element insertion previously described for *v ^1^* (Searles & Voelker, 1986). 20/21 candidate SNPs were either synonymous in nature or resided within non-coding regions. All of these were specific to the *v ^1^* and *v ^+^* chromosomes and therefore we did not consider these further. This left just one remaining candidate SNP, a T>A missense mutation at position chrX:10,924,830. This candidate variant was specific to *v ^24^* chromosomes and would result in a Phe>Ile amino acid change at position 206 along the V polypeptide.

To validate our findings, we performed a literature search for other *v* mutations to see if the same mutation we identified for *v ^24^* (recovered from Gans et al., 1975) has been recovered from an independent screen. To our knowledge, there have been at least 12 independent screens for *v* mutations utilizing different chemical mutagens (Aguirrezabalaga et al., 1995b, 1995a; Ballering et al., 1994, 1997; Nivard et al., 1992, 1993, 1996; Pastink et al., 1989, 1991; Sierra et al., 1993; Vogel & Nivard, 1998; Sierra et al., 2001). From all these screens, there was a total of 260 individual missense mutations resulting in a *v ^–^* phenotype. One of these screens, Nivard et. al, 1993, recovered a *v ^–^* allele ( *v ^275^* ) with the identical *v ^24^* T>A mutation at position chrX:10,924,830 after treating flies with the chemical mutagen methanesulfonate. This is strong corroborating evidence that the mutation we identified for *v ^24^* is indeed causal for the observed *v ^–^* phenotype.

The *v* gene has a predicted tryptophan 2,3-dioxygenase protein domain spanning amino acid positions 16-357 which is required for the production of brown pigments in the eye (Öztürk-Çolak et al., 2024; Searles & Voelker, 1986). The Phe>Ile amino acid switch we identified at position 206 in the *v ^24^* allele would reside within this predicted domain. There is also a predicted ɑ-helix spanning residues 206-226 (Öztürk-Çolak et al., 2024) which could potentially be disrupted with the *v ^24^* amino acid substitution.

A Phe>Ile amino acid change within a protein is a relative conservative substitution since both amino acids contain hydrophobic side chains. Phe contains an aromatic ring while Ile contains an aliphatic side chain. We looked at the predicted Alphafold structure (Jumper et al., 2021) at position 206 and found that the Phe aromatic side chain was orientated towards a pocket of other aromatic side chain containing amino acids ( Figure 1C ). Specifically, this pocket additionally consisted of aromatic amino acids Trp (position 207), Phe (210), Phe (281), and Tyr (282). Pockets of aromatic containing amino acids are commonly referred to as π-π stacking interactions and have been described to promote strong non-covalent stability to proteins (Chen et al., 2018). Loss of an aromatic side chain might destabilize these favorable π-π interactions within this region. If the aromatic interactions within this region are indeed important for V protein stability, we reasoned that mutations disrupting other aromatic amino acids within this pocket would have also been recovered from previous screening efforts. From the 260 missense mutations recovered for *v* loss-of-function, a Phe>Ile ( *v ^286^* , Nivard et al., 1993) and Phe>Ser ( *v ^407^* , Nivard et al., 1996) at position 210 have independently been recovered. These results suggest that this aromatic pocket is likely sensitive to the loss of aromatic containing amino acids, even if the amino acid substitutions remain hydrophobic in nature.

In summary, the T>A missense mutation resulting in a Phe>Ile amino acid change is specific to *v ^24^* and has also been previously shown to result in a *v ^–^* phenotype (Nivard et al., 1993), therefore this mutation is likely resulting in the same *v ^–^* phenotype for the *v ^24^* allele.

## Methods

To prepare genomic DNA-seq libraries for all alleles except *y ^1^* *cv ^1^* *v ^1^* *f ^1^* , genomic DNA from 30 homo/hemizygous flies of the indicated genotypes were extracted using the Qiagen DNeasy Blood and Tissue Kit (Qiagen, Hilden, Germany) utilizing the manufacturer’s insect protocol. Purified genomic DNA was measured with Quant-iT PicoGreen dsDNA Assay Kit (ThermoFisher Scientific, Waltham, MA) and 250 μL of 20 ng/μL DNA in TE buffer with 40 mg of 212-300 μm acid-washed glass beads (Millipore Sigma, Burlington, MA) were sonicated for 10 cycles (30 seconds on, 30 seconds off) in a Bioruptor Pico sonication device (Diagenode, Liege, Belgium) at 4°C. 5 ng of genomic DNA for each sample was then utilized with the NEBNext Ultra II DNA Library Prep Kit for Illumina (New England Biolabs, Ipswich, MA) and completed according to the manufacturer's protocol. Genomic DNA-seq library concentrations were then measured with a Quant-iT PicoGreen dsDNA Assay Kit, pooled, and then 50 nucleotide paired-end DNA sequencing was performed on an Illumina NovaSeq 6000 system using the XP workflow (Illumina, San Diego, CA). For *y ^1^* *cv ^1^* *v ^1^* *f ^1^* , DNA-seq libraries were prepared as previously described (Hammond et al., 2020).

In order to determine mutations at the *v* locus, DNA-seq reads for each genotype ( *fs(1)A1459 ^1^* *v ^24^* , *y ^1^* *cv ^1^* *v ^1^* *f ^1^* , *w ^1^* *Sxl ^F9^* *ct ^6^* , *w ^1^ Sxl ^F2593^ ct ^6^* , *y ^1^ Sxl ^F2^* , *ovo ^D3^ v ^24^* , and *Sxl ^K1274-1^* *v ^24^* ) were mapped to the BDGP Release 6 Drosophila Genome (dos Santos et al., 2015) using Hisat2 (Kim et al., 2019). Mapped reads were then sorted and converted to bam files with Samtools (Li et al., 2009). In order to increase the efficiency of analysis without a super computer, we utilized ChatGPT (all uses of ChatGPT were with version 3.5 by OpenAI) by entering the code and using the query, “How can I optimize this code: bcftools mpileup -f reference.fa alignments.bam | bcftools call -mv -Ob -o calls.bcf”, then adjusting the resulting code to ensure accuracy for the given project. This allowed us to use optimal variant calling parameters for precise variant filtering through Bcftools (Danecek et al., 2021), leading us to only call a decisive variant at a specific position that was consistent among all reads for each respective genotype. Each variant's effect on *v* was annotated and predicted with SnpEff (Cingolani et al., 2012), then stringently filtered through Vembrane (Hartmann et al., 2023) and cross-checked with IGV genomics viewer (Robinson et al., 2011).

Sequencing reads from *w ^1^* *Sxl ^F2593^* *ct ^6^* DNA-seq libraries showed heterozygosity for the *v ^24^* missense mutation (4/8 reads). Since there was not a consensus of reads for either the *v ^24^* mutation or wild type reference nucleotide, we performed Sanger sequencing of this region to determine the exact nucleotide sequence at this position. Two *w ^1^* *Sxl ^F2593^* *ct ^6^* males were crushed with a pestle in Squish Buffer (1M Tris, 0.5M EDTA, 5M NaCl) with Proteinase K (final concentration is 0.4 mg/mL) added. The solution was then left on a heat block at 37°C for 30 minutes and then ramped up to 98°C for 10 minutes. 1 μL of gDNA was then used for a PCR reaction to amplify the region containing the putative *v ^24^* mutation with the following forward (TGTCAAGCGACTGAACCGAG) and reverse (GCACTGCCGGATCAAAGATG) primers. This PCR product was then sent for sequencing to Plasmidsaurus (Louisville, Kentucky). Sanger sequencing confirmed that this region in *w ^1^* *Sxl ^F2593^* *ct ^6^* flies matched the reference genome.

The raw sequencing data are available on the SRA: SRR34448911-SRR34448917.

## Reagents

*fs(1)A1459 ^1^* *v ^24^* BDSC #4318, FBst0004318

*y ^1^* *cv ^1^* *v ^1^* *f ^1^* Recombinant from BDSC #1515, FBst0001515

*w ^1^* *Sxl ^F9^* *ct ^6^* BDSC #58488, FBst0058488

*w ^1^ Sxl ^F2593^ ct ^6^* BDSC #58749, FBst0058749

*y ^1^ Sxl ^F2^* BDSC #4593, FBst0004593

*ovo ^D3^ v ^24^* BDSC #1326, FBst0001326

*Sxl ^K1274-1^* *v ^24^* BDSC #4554, FBst0004554
